# Invitro antibacterial activity of bark, leaf and root extracts of combretum molle plant against streptococcus equi isolated from clinical cases of strangles in donkeys and horses

**DOI:** 10.1186/s12917-024-03954-8

**Published:** 2024-03-13

**Authors:** Ayechew Yetayeh Emiru, Fekadu Regassa, Bojia Endebu Duguma, Asmamaw Kassaye, Belay Desyebelew

**Affiliations:** 1 School of Veterinary Medicine, Woldia University, North Wollo, Ethiopia; 2 College of Veterinary Medicine and Agriculture, Addis Ababa University, Bishoftu, Ethiopia; 3 Donkey sanctuary Ethiopia, Addis Ababa, Ethiopia; 4 Dire Dawa University, Dire Dawa, Ethiopia

**Keywords:** Antibacterial, Combretum molle, In vitro, Strangles, Streptococcus equi

## Abstract

**Background:**

Effective therapy for many infections is becoming difficult due to the evolutionary development of drug resistance, and hence, the development of alternative treatment options mainly from herbs is crucial. The objective of this study was to investigate the antibacterial effects of ethanol extracts of stem bark, leaves and roots of Combretum molle against Streptococcus equi isolated from clinical cases of strangles using in vitro tests.

**Methods:**

Plant extraction was performed using a maceration technique with 80% ethanol. The mean zone of inhibition was determined using the agar well diffusion method. Six serial dilutions with different concentrations (10%, 5%, 2.5%, 1.25%, 0.625% and 0.3125%) of each plant extract were prepared using dimethyl sulfoxide (DMSO). A modified agar microdilution method was used to determine the minimum inhibitory concentration (MICs) of the extracts.

**Results:**

The results revealed that all plant extracts showed significant antibacterial activity. The root extract showed the best antibacterial effect compared to the others at all concentrations, with MZI values of 27.5, 23.225, 20.5, 17.9, 15.65 and 12.25 for the respective concentrations mentioned above and an MIC of 250 µg/ml. It was followed by the stem bark extract, which had MZI values of 24.67, 22.35, 18.225, 16.175, 11.125 and 8.2 millimeters and an MIC of 375 µg/ml. The leaf extract also had significant activity, with MZI values of 20.175, 18.25, 15.7, 13.125, 9.4 and 6.75 in millimeters and an MIC of 500 µg/ml. There was a direct relationship between the concentrations of the plant extracts and the level of inhibition.

**Conclusion:**

The test plant extracts were compared with the conventional antibiotic penicillin G, and the results indicated that the parts of the test plant have significant antibacterial activity, which may support traditional claims and could be candidates for alternative drug discoveries.

## Background

One of the most common respiratory diseases in equine is strangles, which is caused by the bacterium *Streptococcus equi* [1]. The upper respiratory tract and the head and neck lymph nodes are usually infected by the bacteria. It is a globally widespread infectious disease in horses that is economically important and frequently diagnosed [2]. Asymptomatic carrier horses are most likely the primary cause of recurrent infections. A horse can contract a strangles by direct contact with an infected horse or indirectly through contact with contaminated tack, feed, water buckets, brushes, or other items [3]. The disease causes substantial economic losses to the equine industry worldwide, directly due to treatment for prolonged duration, extended recovery period and other serious complications and indirectly because of limitation of horse movement and cancellation of equestrian events [4]. Animals with strangles should be treated with antibiotics containing penicillin [5]. But the organism is now developing resistance for antibiotics. For instance a study Joana D Fonseca et al. (2020) 12.5% of *S. equi* isolated from upper respiratory tract samples were developed resistance to penicillin [6]. Antibiotic resistance is currently a global issue that negatively affects antibiotic therapy, making empirical therapy much more difficult to achieve. Selection for organisms with an improved capacity to survive and procreate in the presence of a drug leads to the evolutionary process that gives rise to drug resistance [7].

Despite their critical role, conventional veterinary services are not widely available in developing countries, and the cost of care and the emergence of antibiotic resistance continue to limit the utility of modern pharmacotherapy [8, 9]. For this reason, livestock farmers especially in rural areas, often turn to traditional healers for remedies for their sick animals. It would be attractive to develop an affordable, socially acceptable and successful treatment that can support contemporary medicine [10].

Research into novel and innovative plant-based antimicrobial agents is one tactic to mitigate the current circumstances [11]. Numerous bioactive compounds occur in plants, most of which likely evolved as chemical defenses against infections or predators [12]. Any plant that has one or more parts, contains compounds with therapeutic value, or serves as a starting point for the synthesis of useful medicines is considered a medicinal plant [13]. For both the human and animal populations in Ethiopia, herbal remedies remain the most important and sometimes the only therapeutic source. It is estimated that between 6,500 and 7,000 species of higher plants are of medicinal importance; of these, 12% are endemic to Ethiopia and used as medicinal plants [14].

### Test organism

*Streptococcus equi* is a gram-positive coccoid bacterium that typically appears in pairs or chains. Colonies cultured on blood agar plates are often mucoid, honey colored, and surrounded by a wide zone of beta hemolysis. *S. equi* colony morphology can be identical to that of S. zooepidemicus, and it is often differentiated by its inability to ferment lactose and sorbitol [15]. *Streptococcus equi* causes strangles in horses. This highly contagious disease is characterized by abscessation of lymph nodes in the head and neck, and leads to high morbidity and occasional mortality [2].

### Plant used for the study

*Combretum molle* (“Yeqola Abalo” in Amharic), known by the common name Velvet Leaved Combertum. The plant belongs to the Combertaceae family, which includes small deciduous trees with a maximum height of 2 to 15 m [16]. In traditional Ethiopian medicine, extracts from the stem bark of *C. Molle* are used to treat liver disease [17]. The plant’s stem bark is used to make “Woiba” in northern Ethiopia and is also said to cure wounds, tuberculosis and malaria [18, 19]. Respiratory diseases in Equines are treated in central Gondar, where the plant is collected, with root extracts and smoke from the stem bark. In addition, the stem bark is used for smoking various utensils, especially for storing milk and the local beer “Tella.” The leaves are also used to clean these utensils.

Phytochemical screening studies of the plant revealed the presence of alkaloids, flavonoids, phenolic compounds, polyphenols, terpenoids, tannins, coumarins, saponins, phytosterols, gums, mucilage, carbohydrates, amino acids and proteins [20, 21]. Studies focusing mainly on the antibacterial and antifungal properties of the plant’s stem bark extract (19, 22, 23, and 24) and antibacterial and antifungal properties of the leaf extracts have demonstrated the plant’s potent antibacterial and antifungal properties. The stem bark leaves and roots of the plant were the plant parts used in this study to evaluate the antibacterial activity of the plant. The plant was selected due to the traditional use of the plant and other scientific works conducted mainly on its antibacterial and antifungal effects.

The aim of this research was to investigate the antibacterial activity of ethanol extracts from steam bark, leaves and roots of *Combretum molle* against *Streptococcus equi* isolated from clinical cases of strangles.

## Materials and methods

### Study area

The plant material was collected from Gondar Zuriya district in Central Gondar, Amhara Regional State, Ethiopia. The area is 698 km from Addis Ababa. Geographically, it lies between 12^0^ 23’ 53” to 12^0^ 30’ 49” N Latitude and 37^0^ 33’ 39” to 37^0^ 37’ 14” E Longitude. The two major soil types are Red soil (Nitosol) and Black soil (Vertisol) [25]. Gondar Zuria District falls in to three agroecological zones is located at 1107–3022 m a.s.l. The two agroecology zones, Dega (2300–3200 m a.s.l.) and Weynadega (1500–2300 m a.s.l) constitute the largest area coverage as compared to the Kolla that falls in the range of 500–1500 ma.s.l. The temperature in the district ranges between 14 and 20 °C with the mean annual temperature of 17.9 °C. Rainfall ranges between 1030 and 1223 mm with the mean annual rainfall of 1100 mm. Land use data of the area indicates; Crops cover 56.5% the area, pasture 14.7%, forests and shrubs 10%, settlements 5.3% and the rest 13.5% is a miscellaneous land [26]. The experimental study of antibacterial activity was carried out in the laboratory of Veterinary Pharmacology, Addis Ababa University College of Veterinary Medicine and Agriculture, Bishoftu, Ethiopia.

### Study design

The study design was experimental and relied on the determination of the in vitro antibacterial effects of ethanol extracts of the bark, leaf and root of *Combretum molle* against *Sreptoccocus equi* isolate.

### Isolation and identification of *Streptococcus equi*

*Streptococcus equi* was isolated from clinical cases of strangles in donkeys and horses presented at Addis Ababa University College of Veterinary Medicine and Agriculture (AAU, CVMA), Donkey Health and Welfare Project (DHWP) and Society for the Protection of Animals Abroad (SPANA) clinics. The samples were collected from nasal swab and pus from unruptured swollen lymph nodes. Isolation and identification of the bacterium was based on cultural examination, microscopic morphology and biochemical reaction. Gram’s stained smears revealed Gram positive cocci which were arranged in pairs and short chains. Direct cultivation of collected samples on modified Edward’s medium as selective medium for *St. equi.* And growing colonies were cultured on blood agar medium and aerobically incubated at 37^o^C for 24–48 h. On blood agar colonies showed small, circular, translucent, glistening colonies with beta hemolysis. There was no growth on Mc-Conkey’s medium. The fermentation tests revealed that the isolates were positive for maltose and negative for lactose, sorbitol and trehalose and negative for catalase and oxidase tests [27, 28].

### Plant collection, authentication and pre-extraction preparation

Parts of the plant were collected from communal land of Gondar Zuriya District, Central Gondar, Ethiopia. Permission to collect the plant material was obtained from Land Administration and Forest Protection Departments of the district. Samples of the plant were authenticated by the National Herbarium of Ethiopia, Addis Ababa University College of Natural Science. Collection was done on March, 2021. The identification was carried out by the senior botanist, Mr. Melaku Wondaferash and the report contained the local name, botanical name and its family; Yeqola Abalo (Amharic); *Combretum molle;* Combertaceae and specimens were deposited in the herbarium with fixed voucher numbers (FR1) in the herbarium. After collection, the plant parts were washed with tap water to remove debris and other unnecessary particles. The samples were then dried at room temperature and ground mechanically with a grinder. The material was then sieved and weighed before maceration.

### Crude extract preparation

Twenty-five grams of grounded and sieved powder from each part of the test plant was weighed using a sensitive balance, placed in a bottle and mixed with 250 ml of 80% ethanol. For sufficient maceration of the plant parts, ratios of 1:5 were used and an orbital shaker was used at maximum speed (160 rpm) for 72 h of mixing. Each sample was then strained with a strainer to remove the solids after 72 h. Whatman filter paper No. 1 was used for additional straining to get a solids-free solution. After the solvent was removed, the solution was concentrated using a vacuum rotary evaporator. The initial weights of the clean Petri dishes were measured using a sensitive balance and labeled. After the concentrated plant extracts were added to these Petri dishes, the remaining solvent was evaporated by heating the extracts at 40 °C in a hot air oven for 48 h. After recording the total weights, the weight of the extracts was calculated by subtracting the initial weight from final weight of the Petri dish. The concentrated plant extracts were then stored in a desiccator until their antimicrobial activity was examined [29].

### Preparation of antibacterial solutions from plant extracts for the in vitro experiment

The in vitro antibacterial effects of the plant extracts were determined by using the agar well diffusion method. Six serial dilutions with different concentrations (10%, 5%, 2.5%, 1.25%, 0.625% and 0.3125%) of each plant extract were prepared using 10% dimethyl sulfoxide (DMSO) [30, 31]. In the first test tube 2 ml of stock solution (10%) was added and each of the remaining five tubes was filled with 1 ml of DMSO. 1 ml of 10% solution from the first tube was transferred to a second test tube to prepare 5%. The procedure continues by transferring 1 ml of solution from the 5% preparation to a third test tube to get a 2.5% concentration, and the procedure continued in a similar manner until a 0.3125% concentration is reached. In transferring from one concentration to another mixing were done by vortex mixer.

### Antibacterial activity test

The agar well diffusion method was used to perform the antibacterial susceptibility test. To check their sterility, Muller-Hinton agar plates were prepared and incubated at 37 °C for 24 h. Plates were considered sterile and were used for antimicrobial susceptibility testing if there was no growth after 24 h. Wells with a diameter of six millimeters were punched into the agar surface at a distance of 20 mm and 15 mm from the plate edges. The purpose of the wells was to store the plant extract solution at any concentration. Using a wire loop, the tops of four to five well-isolated colonies with identical morphology were removed from the nutrient agar, mixed with sterile normal saline, and vortexed. By comparing the turbidity of the bacterial suspension to the McFarland turbidity standards of 0.5, the clarity became more apparent. The McFarland turbidity standard was prepared by combining 9.95 mL of 1% sulfuric acid (0.036 Nb H2SO4) with 0.05 mL of a 1.175% aqueous solution of barium chloride (0.048 N BCL2H2O) [32].

To achieve uniform inoculations, a sterile swab was dipped into the standardized bacterial suspension and streaked over the entire surface of the agar in three different directions. Finally, the swab was wiped over the agar around the edge of the Petri dish. Wells with a diameter of 6 mm were drilled in to the medium using a sterile crock drill and labelled accordingly. Then, using a sterile micropipette, 50 µl of extract solutions of the test plant of each concentration were added to the correspondingly labeled agar wells [33, 34]. Penicillin G served as a positive control in this study, while DMSO served as a negative control. The plates were incubated at 37 °C for 24 h and then stored until the additional extract solutions had diffused into the medium. The diameter of the zone of inhibition was measured in millimeters using a digital calliper [35].

### Minimum inhibitory concentrations (MICs)

The MICs of extracts were determined using a modified agar microdilution method [36]. As mentioned previously, the McFarland standard was established for the bacteria (1.5 × 10^8^ colony forming units (CFU) cells/mL. Each bacterial strain was cultured with MHA supplemented with different concentrations of medicinal plant extracts. To show that no bacterial colonies grew, a control plate label “before” without inoculum and extract was used. Two control plates One labeled “before” (without extract and inoculum) to show that bacterial colony was not grew and the second labeled “after” (without extracts but after inoculation) to confirm that bacteria colonies were present after inoculated and incubated were used. Plates including controls were incubated at 37 °C for 24 h. Accordingly, observations were documented. The minimum inhibitory concentration (MIC) is the lowest concentration of plant extracts that completely suppresses colony growth. A low minimum inhibitory concentration (MIC) was produced by highly active antimicrobial agents, whereas antimicrobial agents with low activity had a high MIC [23].

### Data analysis

The obtained data were coded and entered into a Microsoft Excel spreadsheet, and data analysis was done by using IBM SPSS statistics 20. Descriptive statistical methods (mean and standard error of the mean) were used to summarize the results. One way ANOVA followed by Tukey’s multiple comparison tests was performed to determine statistical significance. P values less than 0.05 were considered significant.

## Results

### The mean zone of inhibition

Each extract of the plant parts was tested at different concentration levels (10%, 5%, 2.5%, 1.25%, 0.625%, and 0.3125%), and the mean zone of inhibition (MZI) was determined at each concentration level by taking the diameter of the zones of inhibition in millimeters (mm) at four isolates of the targeted bacterium (Tables 1, 2 and 3).

**Table 1:** Zone of inhibition (mm) exhibited by the stem bark extract of *Combretum molle* against *Streptococcus equi* (the mean values are the mean ± standard deviation)

Isolate	Zone of inhibition at different concentrations					
	10%	5%	2.5%	1.25%	0.625%	0.3125%
**1**	24.76	22.3	17.8	15.7	10.4	8.7
**2**	25.5	23.5	18.5	16.8	12	9
**3**	25	22	19	16.4	11.5	7.8
**4**	23.4	21.6	17.6	15.8	10.6	8
**Mean**	**24.67 ± 0.8979**	**22.35 ± 0.8185**	**18.225 ± 0.6448**	**16.175 ± 0.5188**	**11.125 ± 0.7544**	**8.2 ± 0.5678**

**Table 2:** Zone of inhibition (mm) exhibited by the leaf extract of *Comretum molle* against *Streptococcus equi* (the mean values are the mean ± standard deviation)

Isolates	Zone of inhibition at different concentrations					
	10%	5%	2.5%	1.25%	0.625%	0.3125%
**1**	20.5	18.5	16	14	10.6	7.2
**2**	19.2	17	15	13.5	8	6
**3**	21	19	16	12	9	6.7
**4**	20	18.5	15.8	13	10	7
**Mean**	**20.175 ± 0.7675**	**18.25 ± 0.8660**	**15.7 ± 0.4761**	**13.125 ± 0.8539**	**9.4 ± 1.1431**	**6.75 ± 0.5252**

**Table 3:** Zone of inhibition (mm) exhibited by the root extract of *Comretum molle* against *Streptococcus equi* (the mean values are the mean ± standard deviation)

Isolates	Zone of inhibition at different concentrations					
	10%	5%	2.5%	1.25%	0.625%	0.3125%
**1**	27	23	20	18.2	16	12.5
**2**	29	24.9	21	19	15.6	11.5
**3**	26	22	20	17.4	15	12
**4**	28	23	19.5	17	16	13
**Mean**	**27.5 ± 1.2909**	**23.225 ± 1.2121**	**20.5 ± 0.6291**	**17.9 ± 0.8869**	**15.65 ± 0.4725**	**12.25 ± 0.6455**

**Fig. 1:**
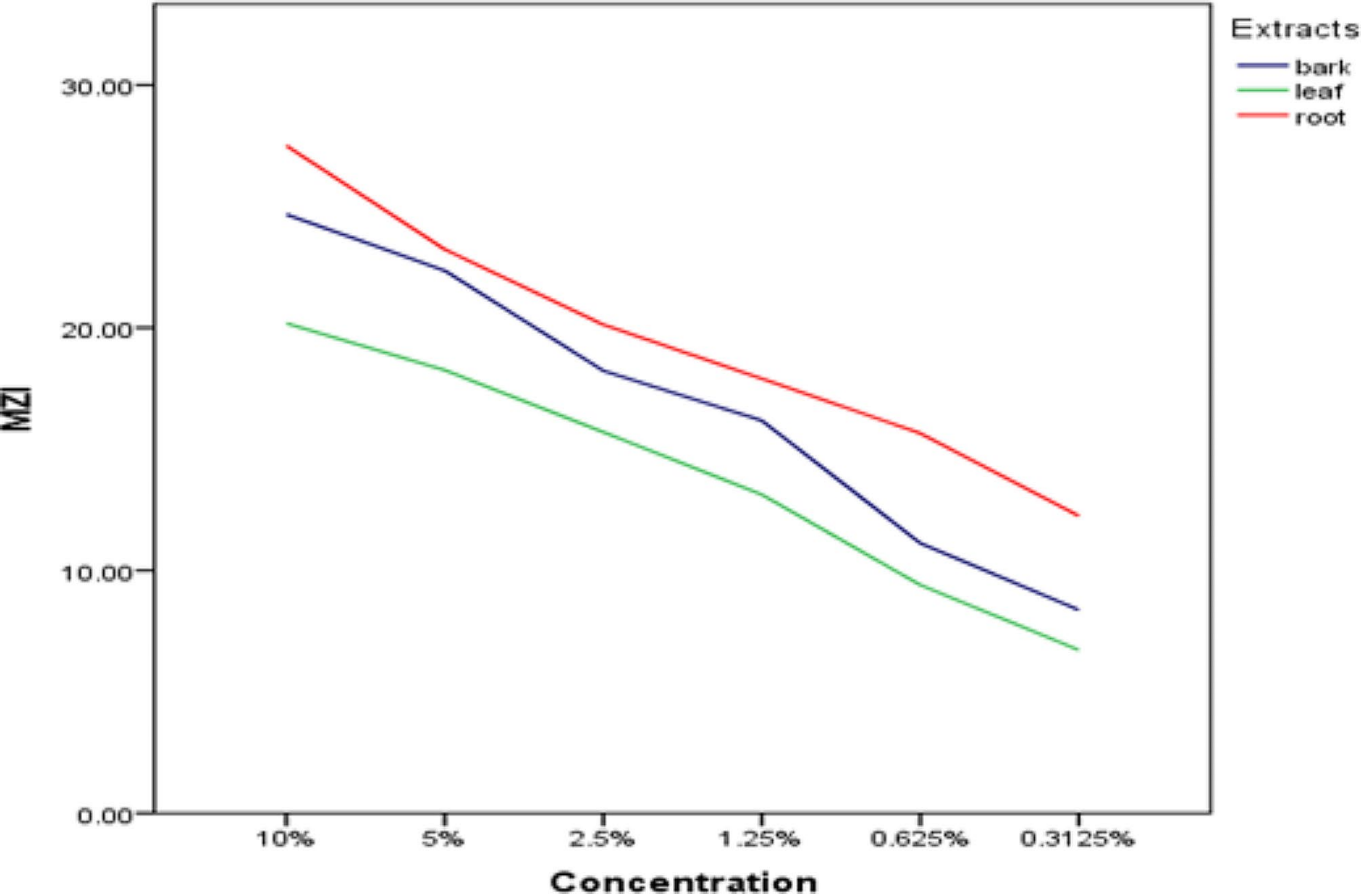
The concentration of the extract and the mean zone of inhibition. (MZI = mean zone of inhibition)

### Comparison with the conventional antibiotic

The mean zone of inhibition of 10% of the extracts was compared with that of the conventional antibiotic penicillin G and recorded (Table 4).

**Table 4:** Comparison of the mean zone of inhibition of 10% extracts with penicillin G

Type of trials	Mean zone of inhibition or MZI (mm)
Bark extracts	24.6
Leaf extract	20.17
Root extract	27.5
Penicillin G	24.5
DMSO	NI

(NI = no inhibition)

### Minimum inhibitory concentrations

The MIC values were determined by the abovementioned method. If the extract showed significant retardation of the growth of the bacteria before inhibition and total absence of visible growth, its MIC was calculated as the mean of the concentration at which the bacterial growth was reduced and the concentration at which no bacterial growth was observed (Table 5).

**Table 5:** MIC of the plant extracts

Extracts	Growth on different concentration in µg/ml						
	15.6	31.25	62.5	125	250	500	1000
Bark	+	+	+	+	±		-
Leaf	+	+	+	+	+		-
Root	+	+	+	+	-	-	-
Control**1**	-	-	-	-	-	-	-
Control **2**	+	+	+	+	+	+	+

+ Growth, ± reduced growth, - no growth

## Discussion

Despite the fact that a large number of antimicrobial agents have been identified, pathogenic microorganisms are constantly developing resistance to them. To find safer and more effective treatments against infectious diseases, recent efforts have been made to explore traditional knowledge [37]. An important source for the development of novel antimicrobial agents is plant life. In vitro antibacterial activity testing is the first step toward achieving this goal [38]. In this study, the antibacterial activity of ethanol extracts of stem bark, leaf and root of *Combretum molle* were evaluated against *Streptococcus equi* isolated from clinical cases of strangles.

Significant activity against the bacteria was demonstrated by all extracts in a dose-dependent manner, meaning that the level of inhibition dropped with decreasing concentration. This is consistent with the Kinde et al. (2015) [19] report on the impact of *Combertum molle* bark on *Streptococcus agalactiae* and *Staphylococcus aureus*, and revealed a clear correlation between the extract concentration and degree of inhibition.

With an MZI at 10% concentration of 27.5 mm (Fig. 1; Table 3) and a MIC of 250 µg/ml, the root extract exhibited more potent antibacterial activity than the other extracts. This finding was consistent with Alferd Ogao’s (2013) research on the extract’s effectiveness against *Staphylococcus aureus*, which also yielded the same result (0.25 mg/ml) [15]. At a 10% concentration, the stem bark demonstrated improved activity with an MZI value of 24.66 mm, which was higher than the value reported from the result obtained against Streptococcus agalactiae, MZI 19.5 mm [23]. The variation could result from a different species of bacteria or from variations in the sensitivity test procedures. The agar well diffusion was used in this study instead of the previously used agar disc diffusion because we are unsure if our 6 mm plane disc will absorb the required amount of drug extract when preparing the herbal drug discs. When using the agar well diffusion method, we are sure that an appropriate amount of space is found in the well where we pour the needed amount of extract [27].

Minimum inhibitory concentration (MIC) of the stem bark extract was 375 µg/ml, lower than the 800 µg/ml for Staphylococcus aureus that Kaleab (2006) reported [22]. Since nutrient agar was used in the previous study to determine MIC, variations in the MIC values could be caused by differences in the isolates or the methodology used. With MZI values of 10% and MIC values of 20.17 mm and 500 µg/ml, respectively, the leaf extract also demonstrated antibacterial activity. These values were significantly higher than those obtained for Staphylococcus aureus, which Motoni reported as having MIC = 0.28 mg/ml or 280 µg/ml [24]. The variation in the bacterial isolate and the method could also be the cause of the disparity in MIC values. As reported by Darmasu, K. et al. (2020) and Parusnath M., et al. (2023); *C. molle* contains different biologically active phytochemical compounds such as alkaloids, saponins, tannins, flavonoids and steroids so that the antimicrobial activities of plants could be related to these phytochemical constituents (20, 21, and 39).

## Conclusion

Strangles is contagious respiratory disease affect equine species. The disease is caused by *Streptococcus equi*. Currently, drug resistance has made it impossible to treat infectious diseases with antibiotics in an effective manner. It is critical to develop an alternative treatment option that primarily uses herbal remedies. *Combretum molle* is the plant widely used in traditional medicine to treat disease conditions both in humans and animals. The aim of the current study was to investigate the antibacterial activity of ethanol extracts of root, bark and leaves of *C. molle*. All tested extracts have shown significant antibacterial activity against clinical isolates of *Streptococcus equi*. The significant activities of the plant extracts were achieved at their higher concentrations. From the tested parts of the plant he root extract has shown the best activity than the others and followed by bark and root extracts. These findings support the traditional social claims that the plant can be used to treat equine respiratory conditions and suggesting its high potential to be developed as an effective antibacterial drug.

## Data Availability

All data included in the manuscript are the original data obtained from the research. The datasets generated and/or analyzed during the current study are not publicly available but are available from the corresponding author upon reasonable request.

## Abbreviations

AAU: Addis Ababa University
BSAC: British Society for Antimicrobial Chemotherapy
DHWP: Donkey Health and Welfare Project
DMSO: Dimethyl sulfoxide
MHA: Muller-Hinton Agar
MIC: Minimum Inhibitory Concentration
MZI: Mean Zone of Inhibition
SPANA: Society for the Protection of Animals Abroad
SPSS: Statistical Package for Social Science
